# Selective decontamination of the gastrointestinal tract in patients undergoing esophageal resection

**DOI:** 10.1186/1471-2482-10-36

**Published:** 2010-12-16

**Authors:** Franziska Näf, René Warschkow, Walter Kolb, Michael Zünd, Jochen Lange, Thomas Steffen

**Affiliations:** 1 Department of Surgery, Kantonsspital St. Gallen (KSSG), CH-9007, St. Gallen, Switzerland

## Abstract

**Background:**

Selective decontamination of the digestive tract (SDD) to eliminate gram-negative bacteria is still not widely accepted, although it reduces the incidence of nosocomial infections. In a previous retrospective study, a clear benefit to perioperative morbidity, and a reduction in nosocomial infections were found in patients who underwent an esophageal anastomosis. Thus, SDD was applied routinely for esophageal anastomoses. We report the outcome of a cohort of 81 patients who underwent this treatment.

**Methods:**

From 2002, patients who underwent an esophageal anastomosis (esophagojejunostomy) were prospectively recorded. Perioperatively, patients received polymyxin, tobramycin, vancomycin and nystatin by mouth four times a day. Outcome was compared to a control group that was treated before 2002 (68 patients without SDD and 53 patients with SDD). Postoperative morbidity and mortality were assessed.

**Results:**

Between 2002 and 2007, 81 patients who underwent an esophageal anastomosis received SDD. Compared to a retrospective control group, patients with SDD had significantly less pneumonia (OR 0.06 (0.01-0.46), p < 0.001) and lower morbidity (OR 0.16 (0.05-0.49), p < 0.001). Furthermore, fewer anastomotic insufficiencies and complications were found. Similar results were found in the analysis of the patients treated before 2002.

**Conclusions:**

SDD significantly reduces perioperative morbidity and mortality in patients who undergo a distal esophageal anastomosis compared to a historical control group. In patients with an anastomotic leakage, there was a strong tendency of SDD to reduce postoperative mortality.

## Background

After the introduction of selective decontamination of the digestive tract (SDD) in intensive care unit patients in 1984, a reduction in the incidence of nosocomial infections in patients with endotracheal tubes was shown [1]. Prophylactic perioperative SDD to prevent nosocomial infection in patients undergoing an esophageal anastomosis is effective and easy to perform [2]. Topical application of nonresorbable antimicrobial agents to the oropharynx and gastrointestinal tract typically prevents secondary colonization with Gram-negative bacteria, S. aureus and yeast. Only selective antibiotics (both topically and systemically) without anti-anaerobic activity are usually used to maintain the anaerobic intestinal flora. These measures reduce the incidence of perioperative nosocomial infections [3-7]. Furthermore, SDD has been shown to be effective in the prevention of esophagojejunal anastomotic leakage after total gastrectomy and has therefore been proposed as a prophylactic measure [2;8;9]. However, SDD has not yet been widely accepted as standard procedure for patients undergoing an esophageal anastomosis, which represents a group of patients with relatively high risk of perioperative morbidity. Pulmonary complications are the major source of morbidity and mortality after esophageal resection [10]. Approximately 30% of patients who undergo esophagectomies suffer from respiratory complications, and 80% of these complications occur within the first five postoperative days [11].

Several risk factors for pulmonary infections in patients who undergo esophagectomies were identified. Elderly patients and those with chronic obstructive pulmonary disease are at risk for the development of major pulmonary complications that require interventions, such as antibiotic therapy, bronchoscopy or endotracheal intubation. Pneumonia is frequently caused by postoperative aspiration and is the major cause of death in these patients. Minor pulmonary complications that do not require interventional measures occur in almost all patients who undergo an esophageal resection [12].

Leakage of the esophageal anastomosis is another serious and potentially life-threatening complication. Together with postoperative pulmonary infections, anastomotic leakage is a major cause of death after esophageal resection [13].

Based on these findings and experiences with intensive care unit patients, SDD was introduced at our hospital as perioperative prophylaxis in patients undergoing an esophageal anastomoses, in a manner analogous to the approach described in Schardey et al. [2]. The intention was to reduce the incidence of postoperative infections and therefore reduce perioperative morbidity and mortality.

In a previously analyzed retrospective cohort, we showed a reduction in postoperative nosocomial infections in patients with SDD compared to patients without SDD (data not published at the time). Based on these positive results, SDD was routinely implemented as perioperative prophylaxis for all patients undergoing an esophageal anastomosis. Consequently, all patients receiving a total or partial esophagectomy, transhiatal esophagogastrectomy or a total gastrectomy were consistently treated with SDD; data were prospectively recorded to determine the perioperative morbidity and mortality (especially in patients who developed an anastomotic leakage) compared to a retrospective control group.

The aim of this study was to determine whether there was a benefit of SDD to postoperative morbidity and mortality in patients undergoing an esophageal anastomosis. For this purpose, the results of our prospective cohort were compared to the previously analyzed retrospective cohort of surgical patients at our institution who did not receive SDD.

## Methods

Between January 2002 and December 2007, a total of 124 patients underwent elective partial or total esophageal resection at a tertiary referral hospital, and data were prospectively recorded and retrospectively analyzed. All patients undergoing an esophageal anastomosis after either total gastrectomy, transhiatal extended gastrectomy or a Merendino procedure were included in the study [14]. Patients who underwent the following procedures were excluded (n = 43): those who underwent a transthoracic esophagectomy were excluded because they had a cervical anastomosis and no intrathoracic anastomoses; patients having a subtotal gastrectomy with pouch reconstruction were excluded because a gastrojejunostomy was considered to be a different procedure. Additionally, patients undergoing a transmediastinal esophagectomy were excluded to separate proximal esophageal anastomoses from distal esophageal anastomoses because we believe these groups differ in terms of anastomotic leakage. Thus, a homogeneous cohort of 81 consecutive patients undergoing an esophageal anastomosis remained for further analyses.

For SDD, patients received a solution of polymyxin (100 milligrams), tobramycin (80 milligrams), vancomycin (125 milligrams) and nystatin (500 milligrams) by mouth four times a day. This solution was administered at a dose of 10 milliliters by mouth every six hours, and the treatment was continued intraoperatively and postoperatively via a gastric tube. The treatment was started on the morning of the day before surgery (they received at least four doses before surgery) and continued until the seventh postoperative day. All patients received total parenteral nutrition postoperatively for 7 days. On postoperative day 7, a radiological imaging study with oral water-soluble contrast was routinely performed to determine whether anastomotic leakage occurred. Anastomotic leakage was defined as an extravasation of the water-soluble contrast during the radiological study. If the radiological test was normal, the patients were allowed to start oral intake and SDD was stopped on day 7. Total parenteral nutrition was reduced when solid oral intake was started, which was usually on postoperative day 9. Pulmonary infection was diagnosed if two or more of the following clinical signs were present: fever, lung infiltrates on radiological imaging studies, purulent respiratory secretion or a positive culture from respiratory secretions. If a pulmonary infection was diagnosed, it was treated with tazobactam-piperacillin according to the hospital guidelines for the treatment of nosocomial pulmonary infections. All patients with suspected pulmonary infections underwent chest radiography to confirm the diagnosis.

All patients (retrospective and prospective, with or without SDD) received perioperative antibiotic prophylaxis of 2 grams of cefamandole intravenously and 500 milligrams of metronidazole intravenously 30 to 60 minutes before the start of the operation, according to the hospital's standard guidelines for perioperative antibiotic prophylaxis. Antibiotic prophylaxis was repeated intraoperatively if the duration of the procedure exceeded four hours.

The standard procedure for an esophageal anastomosis was an end-to-side esophagojejunostomy using a 25 millimeter circular stapling device. A short, crooked approximately 1-centimeter portion of the jejunum was reconstructed using an ENDO GIA™ universal 45 millimeter stapling device.

The outcome was compared to a historic cohort (n = 121) that was treated from 1995 to 2002 at the same institution. Within this cohort, there were two subgroups: one that was treated with SDD (n = 53) and one that was not (n = 68). The surgeon chose whether or not a patient received SDD. Since July 2002, SDD was routinely used in all patients undergoing an esophageal anastomosis (eligible for this study: n = 81). Age, gender, body mass index (BMI), ASA score, anastomotic leakage, pulmonary infection and mortality rate were compared among the three groups. Perioperative morbidity was analyzed using the classification described by Dindo et al. [15].

All patients provided informed consent to the operative treatment, the administration of SDD and further analyses of data from their medical records. The medical ethics committee approved the analysis of these data.

Statistical analysis was performed using SPSS 11.5 software (SPSS Inc., Chicago, IL, USA). A two-sided p-value < 0.05 was considered statistically significant. Continuous data are expressed as mean ± standard deviation. Confidence intervals (95% CI) of binominal proportions were estimated according to Agresti and Coull. Mann-Whitney U tests and Kruskal Wallis tests were applied to compare continuous data. Chi-Square tests were used to compare proportions.

## Results

### Prospective cohort: Specification of sample

Between 2002 and 2007, a cohort of 81 patients who underwent an esophageal anastomosis (esophagojejunostomy) and received SDD was obtained. A total gastrectomy was performed in 41 patients (51%), transhiatal esophagogastrectomy was performed in 26 (32%) patients and a Merendino procedure was performed in 14 (17%) (14). The mean patient age was 63.1 ± 11.5 years; this cohort included 47 (58%) women.

### Comparability of groups

In Table 1, baseline characteristics of both the prospective and retrospective groups are provided. No significant differences in age, BMI or ASA classification were found between the three groups.

**Table 1:** Baseline characteristics of retrospective and prospective groups

	Retrospective no SDD (n = 68)	Retrospective SDD (n = 53)	Prospective SDD (n = 81)	**p value**^**A)**^
Age *[years]*	63.0 ± 12.1	63.4 ± 13.0	63.1 ± 11.5	0.831

BMI *[kg/m^2^]*	23.9 ± 3.9	24.7 ± 3.7	25.0 ± 4.7	0.469

ASA I	4 (5.9%)	2 (3.8%)	3 (3.7%)	0.296
II	36 (52.9%)	34 (64.2%)	57 (70.4%)	
III	28 (41.2%)	17 (32.1%)	21 (25.9%)	

mean ± standard deviation n (percentage)

^A) ^Kruskal Wallis test for continuous data and Chi-square test for categorical data

### Retrospective analysis (Table 2)

Patients from the retrospective subgroup with SDD were compared to patients who had no SDD. Significantly fewer patients with SDD than those without SDD died within the first 30 postoperative days. A mortality rate of 17.6% (12/68; 95% CI 10.2% - 28.5%) was found in the retrospective subgroup without SDD compared to the mortality rate of 1.9% (1/53; 95% CI 0.3% - 13.5%) in the subgroup with SDD (p = 0.005). Of the patients without SDD, 13.2% (9/68; 95% CI 6.9% - 23.5%) developed an anastomotic leakage, whereas only 3.8% (2/53; 95% CI 0.3% - 13.5%) of the retrospective subgroup with SDD developed an anastomotic leakage (p = 0.072). Of the patients who had no SDD, 25.0% (17/68; 95% CI 16.2% - 36.5%) developed postoperative pneumonia compared to 11.3% (6/53; 95% CI 4.9% - 22.9%) of the patients with SDD (p = 0.057). The postoperative length of hospital stay was shorter in the retrospective subgroup with SDD (21.8 ± 13.7 vs 25.2 ± 18.0 days, p = 0.145). According to the Dindos classification, overall morbidity was less severe in patients with SDD (p = 0.009) (Table 3).

**Table 2:** Outcome analysis

	Group			p value
		
	*retrospective without SDD (n = 68)*	*retrospective with SDD (n = 53)*	*prospective with SDD (n = 81)*	
Mortality [30 days postoperative]	12 (17.6%) (10.2% - 28.5%) OR: 1 ^ref)^	1 (1.9%) (0.0% - 10.9%) OR: 0.09 (0.01-0.71)	1 (1.2%) (0.0% - 7.3%) OR: 0.06 (0.01-0.46)	<0.001 ^A)^
Anastomotic Leakage	9 (13.2%) (6.9% - 23.5%) OR: 1 ^ref)^	2 (3.8%) (0.3% - 13.5%) OR: 0.26 (0.05 - 1.24)	6 (7.4%) (3.2% - 15.5%) OR: 0.52 (0.18-1.56)	0.162 ^A)^
Postoperative Pneumonia	17 (25.0%) (16.2% - 36.5%) OR: 1 ^ref)^	6 (11.3%) (4.9% - 22.9%) OR: 0.38 (0.14-1.05)	4 (4.9%) (1.6% - 12.4%) OR: 0.16 (0.05-0.49)	0.001 ^A)^
length of hospital stay [days]	25.2 ± 18.0	21.8 ± 13.7	20.2 ± 10.9	0.121 ^B)^

n (percentage) (95% CI for percentage) Odds ratio (95% CI for Odds ratio)

^A) ^Chi square tests for overall significance ^B) ^Kruskal Wallis tests for overall significance

Closed testing procedure with chi square tests for: Mortality Leakage Pneumonia

retrospective with SDD - retrospective without SDD p = 0.005 p = 0.072 p = 0.057

prospective with SDD - retrospective without SDD p < 0.001 p = 0.239 p < 0.001

retrospective with SDD - prospective with SDD p = 0.761 p = 0.385 p = 0.169

**Table 3:** Classification of postoperative complications according to Dindo et al. (15)

Dindo classification	Group		
	
	*retrospective without SDD* *(n = 68)*	*retrospective with SDD* *(n = 53)*	*prospective with SDD* *(n = 81)*
grade I	0 (0.0%)	0 (0.0%)	3 (3.7%)
grade II	43 (63.2%)	44 (83.0%)	65 (80.2%)
grade IIIa	6 (8.8%)	3 (5.7%)	5 (6.2%)
grade IIIb	8 (11.8%)	3 (5.7%)	6 (7.4%)
grade IVa	4 (5.9%)	2 (3.8%)	1 (1.2%)
grade IVb	0 (0.0%)	0 (0%)	1 (1.2%)
grade V	7 (10.3%)	1 (1.9%)	0 (0.0%)

Kruskal Wallis test for overall significance: p = 0.002

Closed testing procedure with Mann Whitney U-Tests:

- retrospective with SDD vs retrospective without SDD: p = 0.009

- prospective with SDD vs retrospective without SDD: p = 0.001

- retrospective with SDD vs prospective with SDD: p = 0.819

### Longitudinal analysis

The retrospective subgroup with SDD was compared to the prospective cohort with SDD (Table 2), and no statistically significant differences were found in terms of 30-day mortality: 1.9% (1/53; 95% CI 0.0% - 10.9%) in the retrospective subgroup compared to 1.2% (1/81; 95% CI 0.0% - 7.3%) in the prospective cohort (p = 0.761). In the retrospective subgroup, 11.3% (6/53; 95% CI 4.9% - 22.0%) developed postoperative pneumonia compared to 4.9% (4/81; 95% CI 1.6% - 12.4%) in the prospective cohort (p = 0.169). In the retrospective subgroup with SDD, 3.8% (2/53; 95% CI 0.3% - 13.5%) developed an anastomotic leakage, whereas 7.4% (6/81; 95% CI 3.2% - 15.5%) suffered from anastomotic leakage in the prospective cohort (p = 0.385). The length of postoperative hospital stay did not differ significantly between the prospective cohort and the retrospective subgroup with SDD (20.2 ± 10.9 vs 21.8 ± 13.7 days, p = 0.861).

Patients without SDD were compared to the patients of the prospective cohort with SDD (Table 2); the postoperative 30-day mortality rate was significantly lower in patients with SDD (1.2% vs. 17.6%; p = 0.001). Patients with SDD developed pneumonia postoperatively less often (4.9%; 4/81; 95% CI 1.6% - 12.5%) than patients without SDD (25.0%; 17/68; 95% CI 16.2% - 36.6%; p = 0.001). Fewer patients with SDD than patients without SDD developed an anastomotic leakage (6/81 vs. 9/68; 7.4% vs. 13.2%; p = 0.239). Patients with SDD in the prospective cohort had significantly fewer severe complications than patients without SDD in the retrospective subgroup (p = 0.001) (Table 3). The postoperative hospital stay was shorter in the prospective cohort with SDD than in patients without SDD (20.2 ± 10.9 vs 25.2 ± 18.0 days, p = 0.046).

Pooled subgroup analysis of patients with anastomotic leakage revealed a mortality rate of 55.6% (5/9) in the patients without SDD compared to 12.5% (1/8) in all patients with SDD (p = 0.064).

## Discussion

This observational study corroborates the hypothesis that SDD reduces perioperative morbidity and mortality in patients who undergo a distal esophageal anastomosis [4]. In the present study, perioperative mortality was significantly reduced in patients undergoing a distal esophageal anastomosis when perioperative SDD was performed. Furthermore, significantly fewer patients developed perioperative pulmonary infections. The perioperative mortality rate of patients who postoperatively developed an anastomotic leakage was clearly lower in the SDD group, although this difference was not significant. However, we consider this result to be a strong indicator of the benefit of SDD. Anastomotic leakage is a potentially life-threatening postoperative complication. The mortality rate in patients with anastomotic leakage after total gastrectomy was reported to be as high as 45% [16]. In our patients without SDD, a mortality rate of 55.6% was found, whereas patients with SDD had a mortality rate of 12.5%. Furthermore, our results are comparable to the results of the study by Schardey et al., in which significantly fewer pulmonary infections were described, and a tendency of fewer anastomotic insufficiencies was found in patients receiving SDD [2].

SDD has not been widely adopted as a prophylactic measure in patients undergoing esophageal anastomoses. One possible reason for this might be the fear of complications of such a nonspecific antibiotic treatment. In the studies by Tetteroo et al., no increase in antibiotic resistance was found in patients receiving SDD found [4-7;17]. Additionally, the most recent Cochrane review on antibiotic prophylaxis reported no resistance associated with SDD in one trial [18]. In the study by de Jonge et al., a decreased development of resistance among SDD-treated patients in intensive care over a 27-month period was reported [19]. Although not measured, SDD-associated complications in our patients seldom occur; however, they most often present as nausea, which is considered to be a mild side effect. Additionally, no SDD-associated side effects were reported in a recently published study by Roos et al. [20]. Similar mortality rates and incidence of perioperative infections were found when SDD was employed in patients undergoing elective colorectal surgery compared to patients receiving the same operation but not receiving SDD. However, in that study, SDD was not applied in a standardized fashion, and pulmonary infections were not significantly reduced in the SDD group. We assume that patients have recurrent micro-aspirations after esophagus resection, and therefore, SDD seems to effectively prevent the development of pulmonary infections in our patient cohort.

We are aware of some relevant limitations of this study. The comparison of a prospective cohort with a retrospective cohort is problematic. However, apart from only a few data from the literature that address this specific problem, our retrospective patient cohort is the only source of data available. We aimed to minimize the risk of bias by statistically comparing all groups where no significant differences in the baseline characteristics were found. Furthermore, when comparing the retrospective subgroups with and without SDD, essentially the same outcome was obtained. However, the absence of evidence is not evidence for absence, and longitudinal analyses are always associated with a risk of bias. Furthermore, we assume that advancements in intensive care medicine and anesthesia have occurred over the course of data collection. This possible influence on perioperative morbidity and mortality was not addressed in the current study.

Despite the above-mentioned possible limitations of this study, our results show a strong tendency in favor of SDD for patients undergoing a distal esophageal anastomosis. We encourage institutions where SDD is not performed to conduct a double-blind randomized controlled prospective study to obtain results regarding SDD use with a higher level of evidence than provided here.

## Conclusions

We conclude that SDD in patients undergoing an esophageal anastomosis may provide perioperative morbidity and mortality benefits.

## Competing interests

The authors declare that they have no competing interests.

## Authors' contributions

FN performed the data acquisition and drafted the manuscript. RW participated in the study design and performed the statistical analysis. FN and RW contributed equally to this manuscript. WK performed data acquisition of the retrospective cohort. MZ and JL critically revised the manuscript for important intellectual content. TS conceived the study and participated in the study design, drafting the manuscript and performing the revisions.

All authors read and approved the final manuscript.

## Pre-publication history

The pre-publication history for this paper can be accessed here:

http://www.biomedcentral.com/1471-2482/10/36/prepub
